# Diagnosis and treatment of secretory carcinoma arising from the oral minor salivary gland: Two case reports

**DOI:** 10.1097/MD.0000000000028390

**Published:** 2021-12-23

**Authors:** Masaru Ogawa, Satoshi Yokoo, Takahiro Yamaguchi, Keisuke Suzuki, Mai Seki-Soda, Takahiro Shimizu, Jun Kurihara, Takaya Makiguchi

**Affiliations:** a Department of Oral and Maxillofacial Surgery, and Plastic Surgery, Gunma University Graduate School of Medicine, 4-39-15 Showa-machi, Maebashi, Gunma, Japan; b Department of Diagnostic Pathology, Gunma University Graduate School of Medicine, 4-39-15 Showa-machi, Maebashi, Gunma, Japan.

**Keywords:** ETS variant 6-neurotrophic receptor tyrosine kinase 3 fusion gene, mammary analogue secretory carcinoma, oral cavity, secretory carcinoma

## Abstract

**Introduction:**

Secretory carcinoma (SC) is a malignancy of the salivary glands, which is similar to SC of the breast regarding its association with neurotrophic tyrosine receptor kinase fusion-positive gene. SC is a recently described salivary gland tumor, and there are a few reports describing oral minor salivary gland-derived SC. We reported two cases of SC in the oral cavity and reviewed the literature.

**Patient concerns:**

The patients included a 65-year-old Japanese woman who presented with a mass of the upper lip and an 84-year-old Japanese man who presented with a mass on the buccal mucosa.

**Diagnosis:**

Diagnosis was based on histomorphological and immunohistochemical findings and identification of a specific translocation of the ETS variant 6-neurotrophic receptor tyrosine kinase 3 gene fusion. Case 1 was finally diagnosed using reverse transcription-polymerase chain reaction with formalin-fixed paraffin-embedded tissue samples, while case 2 was diagnosed using fluorescence in situ hybridization analysis.

**Interventions and outcomes:**

In case 1, excisional biopsy was done and there was no recurrence observed in five-year follow-up. In case 2, tumor resection was done and there was no recurrence observed in two-year follow-up.

**Conclusion:**

It is highly likely for many cases of SC to be initially diagnosed as acinic cell carcinoma (AciCC) owing to their similar histological findings. The treatment strategy for minor salivary gland-originated SC is similar to that of AciCC; however, SC is often highly malignant and involves a high risk of cervical lymph node metastasis. Thus, establishing an accurate diagnosis together with pathologists and confirming the presence of the ETS variant 6-neurotrophic receptor tyrosine kinase 3 fusion gene using genetic analysis is important.

## 1 Introduction

In 1996, McDivitt et al reported mammary secretory carcinoma (SC) as a histological subtype of breast cancer.^[1]^ Mammary SC is caused by the ETS variant 6-neurotrophic receptor tyrosine kinase 3 (ETV6-NTRK3) fusion gene through the phosphatidylinositol 3-kinase/protein kinase B and mitogen-activated protein kinase pathways.^[2]^ In 2002, Hirokawa et al noted histological similarities between acinic cell carcinoma (AciCC) of the salivary gland and mammary SC.^[3]^ In 2010, Skálová et al found that the ETV6-NTRK3 fusion gene was expressed in salivary gland tumors that were previously diagnosed as AciCC and proposed the name mammary analogue SC.^[4]^ However, in 2017, the WHO classification of head and neck tumors described it as SC of the salivary gland^[5]^; hence, the name was unified to SC in this report.

SC in the head and neck region develops in individuals in their 40 s, which is a relatively early age of onset compared to that for AciCC; however, a childhood-onset case has similarly been reported.^[4]^ SC showed no sex predilection. Approximately 60–70% of the cases were located in the parotid gland, and the total number of cases in the major salivary glands, including the submandibular gland, accounted for approximately 70–80%.^[6,7]^ Overall, there are a few reports describing minor salivary gland-derived SCs.^[8–10]^ The true frequency of occurrence is unclear because SC is a recently described disease entity, and a few SC cases could have been previously diagnosed as AciCC. Although most SCs are low-grade malignancies, a small subset is reported to be high-grade compared to AciCC,^[11,12]^ differentiation between these carcinomas is important.

We reported two cases of SC in the oral cavity and discussed the grade of malignancy of SC with pooled analysis of the recent literature.

## 2 Case presentation

### 2.1 Case 1

**Figure 1:**
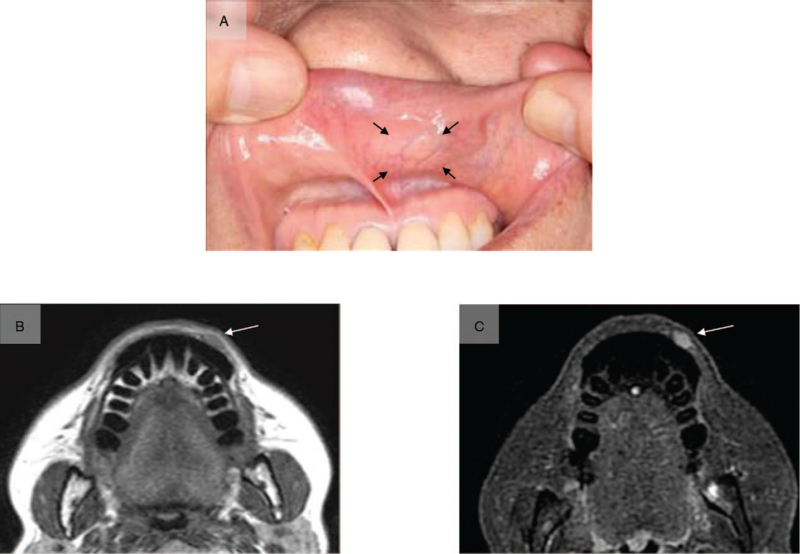
Clinical findings. An elastic-hard protruding mass measuring 15 x 10 mm was noted on the left side of the upper lip (A). The mass exhibited moderate intensity on contrast T1-weighted magnetic resonance imaging (B) and high intensity on short T1 inversion recovery (C). The continuity of the orbicularis oris muscle was retained.

**Figure 2:**
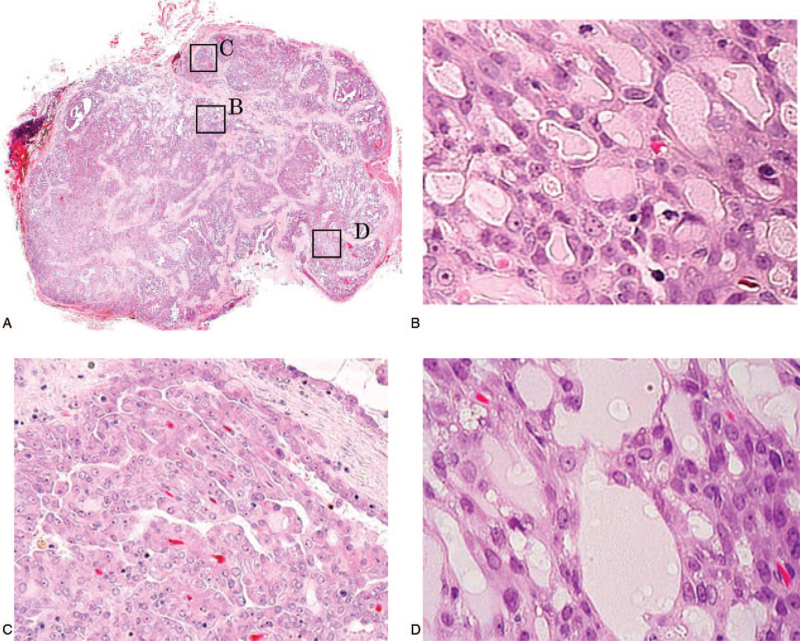
Histological findings (hematoxylin and eosin staining). The lesion was a nodular tumor, and the boundary with the surrounding area was clear, with no evidence of encapsulation (A) (magnification 10 × ). Microcystic (B) (magnification 200 × ), papillary-cystic (C) (magnification 200 × ), and follicular (D) (magnification 400 × ) patterns of tumor cell proliferation were mixed.

A 65-year-old Japanese woman with a two-year history of a gradually enlarging mass on the left side of the upper lip consulted with the Department of Oral and Maxillofacial Surgery, Gunma University Hospital. On clinical examination, a painless, elastic-hard, protruding mass measuring 15 × 10 mm was noted on the left side of the upper lip (Fig. 1A). The overlying mucosa was a flat surface, and the color was normal with no adhesion to the mass. The mass exhibited moderate intensity on contrast T1-weighted magnetic resonance imaging (MRI) and high intensity on short T1 inversion recovery. Additionally, the continuity of the orbicularis oris muscle was retained (Fig. 1B, C). On fluorodeoxyglucose-positron emission tomography, no abnormal accumulation was noted in the cervical lymph nodes or distant organs. These clinical and MRI findings suggested a benign salivary gland tumor. An excisional biopsy was performed, and as the mass was not adherent to the surrounding tissues, dissection was easily performed. Macroscopic observation of the cut surface of the excisional biopsy specimen revealed that the mass was spherical and solid, and the boundary with the overlying mucosa was clear. Histopathological examination revealed that the mass was a 15-mm nodular tumor, and its boundary with the surrounding tissue was clear with no evidence of encapsulation (Fig. 2A). It showed mixed characteristics of microcystic (Fig. 2B), papillary-cystic (Fig. 2C), and follicular (Fig. 2D) patterns of tumor cell proliferation. Polymorphous low-grade adenocarcinoma, AciCC, and SC were considered in the differential diagnosis based on the results of hematoxylin and eosin staining; immunostaining and special staining were performed for differentiation (Table 1).

**Table 1:** Antibodies used for immunohistochemical study.

Primary antibodies	Source	Dilution	Clone	Purpose
CK19	Novocastra	1:100	Mouse monoclonal	• Epithelial cell marker • Develops in some basal cells, staining pattern that is homogenous for the breast malignant tumor
S-100	Dako	1:200	Mouse monoclonal	• Mesenchymal cell marker • Intermediate filament which is common to a mesenchyma system cell
Vimentin	Dako	1:10	Mouse monoclonal	• Mesenchymal cell marker • Intermediate filament which is common to a mesenchyma system cell
Mammaglobin	Dako	1:100	Mouse monoclonal	• Breast cancer specific marker • Develops in breast duct epithelium, an apocrine gland and an eccrine gland epithelium of the normal skin
GCDFP15	Abcam	1:200	Mouse monoclonal	• Breast cancer specific markers • Develops in breast duct epithelium, an apocrine gland of the normal skin
GATA-3	Abcam	1:100	Rabbit polyclonal	• Breast cancer specific markers • GATA familly which is the transcription factor in the nucleus • Expression abnormal for breast cancer, colon cancer
MUC4	Abcam	1:500	Mouse monoclonal	• Membrane-bound mucin • Participate in cell proliferation through the mutual participation with the glycoproteinErb2/HER2 family • Expression abnormal for breast cancer, pancreatic cancer, cholangiocarcinoma, colon cancer

CK19 = cytokeratin 19, GCDFP15 = Gross cystic disease fluid protein 15.

**Figure 3:**
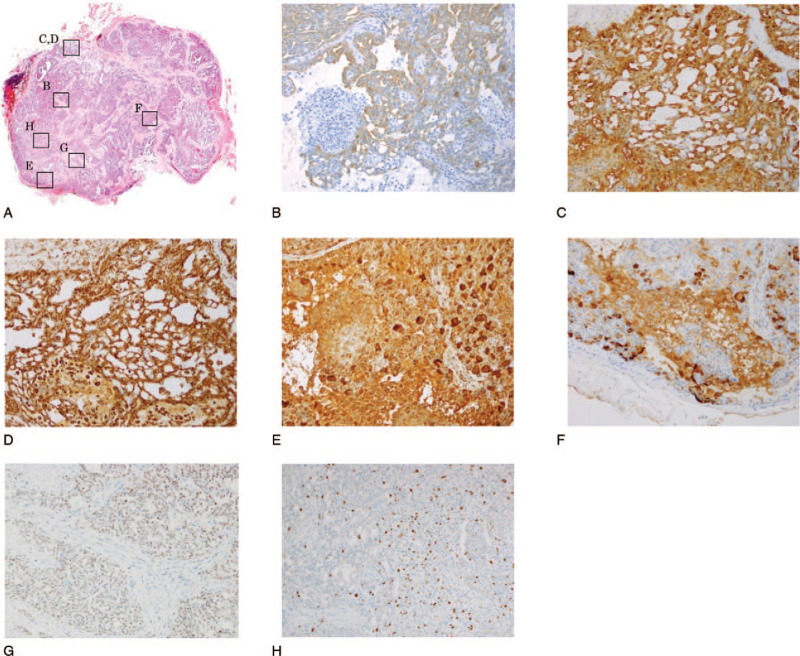
Immunohistochemical findings. Hematoxylin and eosin (A) and immunostaining with Cytokeratin 19 (B), S-100 (C), Vimentin (D), Mammaglobin (E), gross cystic disease fluid protein 15 (F), and GATA3 (G) (magnification 100 × ). An index of tumor cell proliferative activity, the MIB-1 index, was 3% (H) (magnification 100 × ).

**Figure 4:**
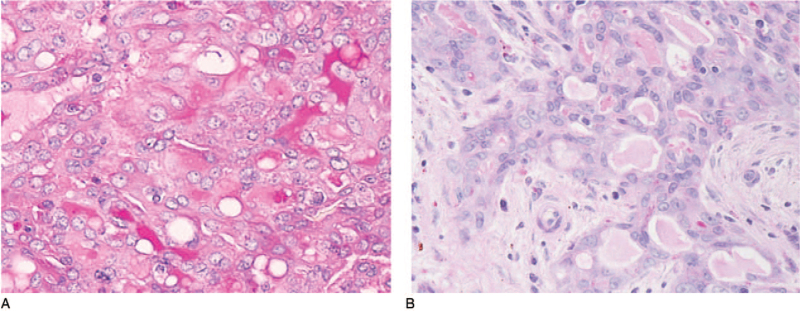
Special staining findings. There were a few periodic acid-Schiff (PAS)-positive granules in the tumor cell cytoplasm (A) (magnification 400 × ). Diastase digestion PAS staining was positive in abundant eosinophilic homogeneous secretions in microcystic and follicular spaces (B) (magnification 400 × ).

**Figure 5:**
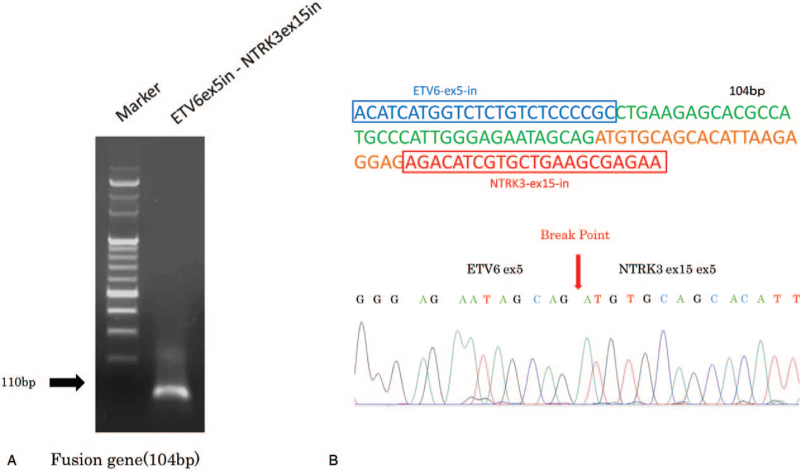
Reverse transcription-polymerase chain reaction (RT-PCR) for the detection of ETV6-NTRK3 fusion gene transcripts. RT-PCR analysis showed amplification of the ETV6-NTRK3 fusion gene (A). Direct sequencing of the amplified RT-PCR product confirmed the presence of ETV6-NTRK3 rearrangement (B).

Immunohistochemistry showed that the tumor was positive for cytokeratin 19 (CK19), S-100, vimentin, mammagloblin, gross cystic disease fluid protein 15 (GCDFP15), and GATA3. These findings are consistent with the immunostaining findings frequently observed in SC.^[4,13–16]^ The MIB-1 index, which indicates tumor cell proliferative activity, was 3% (Fig. 3 A-H). In addition, there were a few periodic acid–Schiff-positive granules in the cytoplasm of tumor cells (Fig. 4A). Periodic acid–Schiff with diastase digestion staining was positive in the abundant eosinophilic homogeneous secretions in microcystic and follicular spaces (Fig. 4B). Furthermore, the examination for ETV6-NTRK3 gene fusion was performed using a formalin-fixed paraffin-embedded tissue sample, and a positive result was obtained in reverse transcription-polymerase chain reaction (Fig. 5A). Direct sequencing of the amplified reverse transcription-polymerase chain reaction product confirmed the presence of ETV6-NTRK3 rearrangement (Fig. 5B), leading to the definitive diagnosis of SC. To ensure a malignant negative margin, additional resection was performed under general anesthesia, and the resection margin was set at 10 mm from the scar of the previous excisional biopsy. No residual tumor tissue was observed in the resected specimen. The tumor was staged pT1 cN0, and adjuvant therapy was not indicated. For five years postoperatively, the patient showed no evidence of recurrence or metastasis.

### 2.2 Case 2

**Figure 6:**
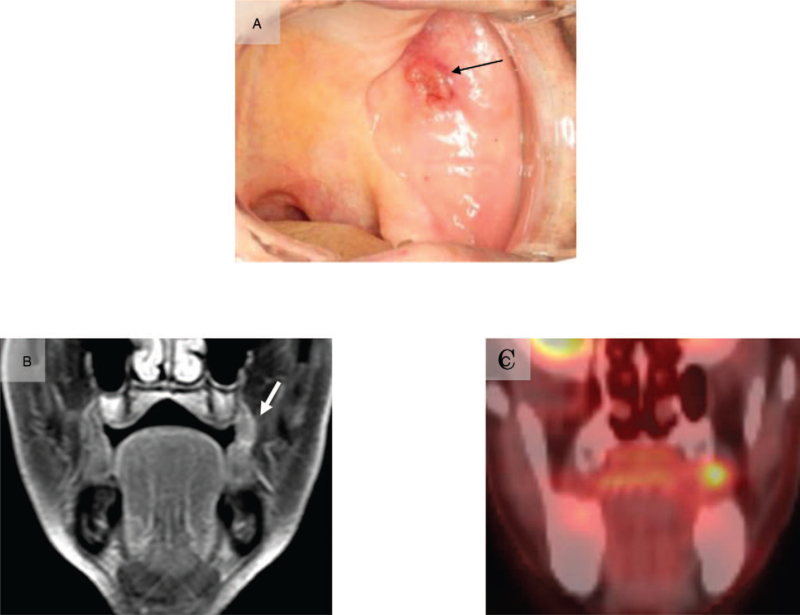
Clinical findings. An elastic-hard mass measuring 17 × 15 mm in size was observed on the left buccal mucosa (A). The mass exhibited moderate intensity on contrast T1-weighted magnetic resonance imaging, and advancement to the buccinator muscle was noted (B). On fluorodeoxyglucose-positron emission tomography (FDG-PET) with an SUVmax of 4.6, FDG accumulation was detected in the left buccal mucosa (C).

**Figure 7:**
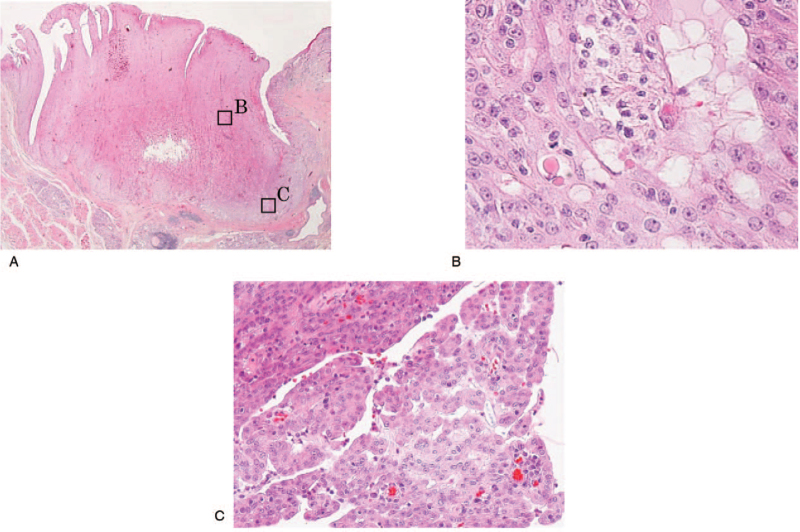
Histological findings (hematoxylin and eosin staining). The lesion was a nodular tumor, and the boundary with the surrounding area was clear, with no evidence of encapsulation (A) (magnification 10 × ). Microcystic (B) (magnification 400 × ) and papillary-cystic (C) (magnification 200 × ) patterns of tumor cell proliferation were mixed.

An 84-year-old Japanese man presenting with a mass on the left buccal mucosa consulted with the Department of Oral and Maxillofacial Surgery, Gunma University Hospital. On clinical examination, an elastic-hard mass measuring 17 × 15 mm was observed on the left buccal mucosa (Fig. 6A). The mass exhibited moderate intensity on contrast T1-weighted MRI, and advancement to the buccinator muscle was noted (Fig. 6B). The fluorodeoxyglucose-positron emission tomography scan revealed that the maximum standardized uptake value (SUVmax) of FDG was 4.6 in the left buccal mucosa (Fig. 6C). There was no evidence of metastasis in the cervical lymph nodes or distant organs. In the biopsy specimen, microcystic and papillary-cystic patterns of tumor cells that were suggestive of AciCC or SC were observed. These clinical, histological, and MRI findings suggested a malignant left buccal mucosal salivary gland tumor. Tumor resection with a 10-mm safety margin was performed under general anesthesia. Macroscopic observation of the cut surface of the surgical specimen revealed that it was white and solid, and the boundary with the surrounding tissues was clear. Histopathological examination showed that it was a 15-mm nodular tumor, and its boundary with the surrounding tissues was clear, with no evidence of encapsulation (Fig. 7A). Furthermore, it showed mixed features of microcystic and papillary-cystic patterns of tumor cell proliferation (Fig. 7B,C).

**Figure 8:**
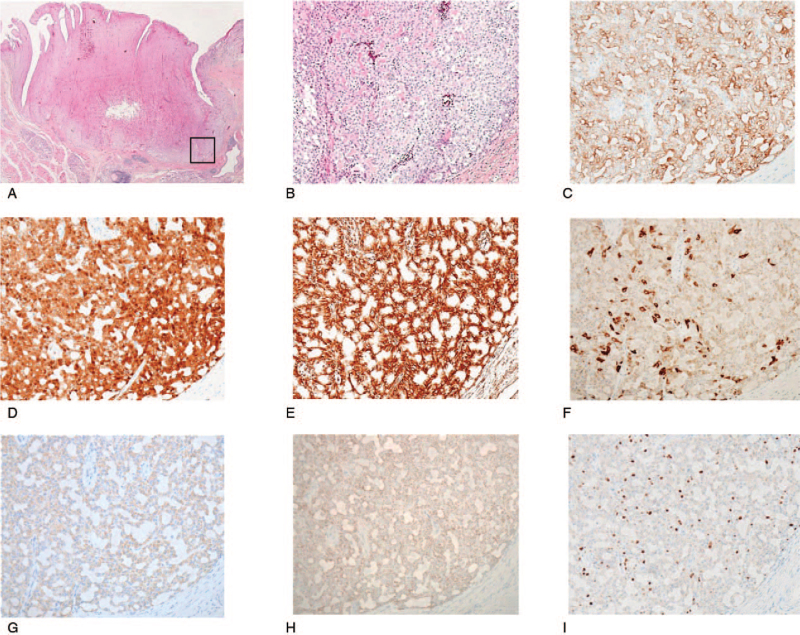
Immunohistochemical findings. Hematoxylin and eosin (A,B) (magnification 10 × , 200 × ) and immunostaining with Cytokeratin 19 (C), S-100 (D), Vimentin (E), Mammaglobin (F), gross cystic disease fluid protein 15 (G), and MUC4 (H) (magnification 200 × ). An index of tumor cell proliferative activity, the MIB-1 index, was 10% (I) (magnification 200 × ).

**Figure 9:**
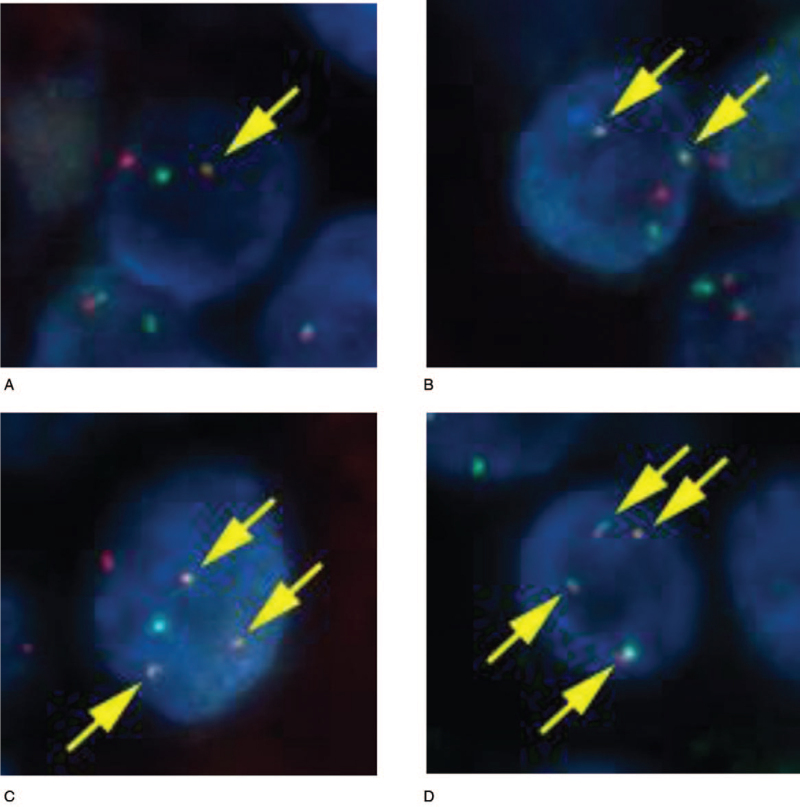
ETV6-NTRK3 fluorescence in situ hybridisation. Signals of the ETV6 and NTRK3 probes are presented in green and red, respectively. The number of fusion gene signals was classified into four patterns: 1 fusion gene signal (A), 2 signals (B), 3 signals (C), and 4 signals (D).

Immunohistochemistry showed that the tumor was positive for CK19, S-100, vimentin, mammagloblin, GCDFP15, and MUC4. These findings are consistent with the immunostaining findings, which are frequently observed in SC (Fig. 8 A-I).^[4,13,17]^ The MIB-1 index was 10%. In addition, genetic analysis was performed using fluorescence in situ hybridization analysis, wherein the ETV6-NTRK3 fusion gene accompanied by chromosomal translocation t(12; 15)(p13; q25) was detected (Fig. 9 A-D). Based on these findings, a definitive diagnosis of SC was established. The tumor was staged pT1 cN0, and adjuvant therapy was not indicated. At two years after surgery, the patient had a good prognosis with no recurrence or metastasis.

## 3 Discussion

Sixty-eight cases of minor salivary gland-originated SC have been reported between 2010 and 2017.^[4,7–11,18–43]^ These cases were identified in a literature search conducted using keywords such as “mammary analogue secretory carcinoma,” “secretory carcinoma”, “oral cavity”, “buccal mucosa,” “lip,” “palate,” “gingiva,” and “tongue” in PubMed and the Japan Medical Abstracts Society databases. Age, sex, location in the oral cavity, size, TNM classification, treatment, metastasis, local recurrence, follow-up period, and survival rate were all described in 21 of the 68 cases. Pooled analysis of 23 cases, which included the two patients of the present case report, was performed (Table 2). Of the 23 patients, SC developed in the lips in nine patients and the buccal mucosa in seven patients, including our patients. These two locations accounted for 70% of all cases. As the labial mucosa is classified as buccal mucosa in the oral cavity category of Union for International Cancer Control classification, the buccal mucosa accounts for approximately 70% of all cases. Histopathologically, tumor cells in SC proliferate in microcystic, papillary-cystic, and follicular patterns.^[4,6]^ However, as this histological morphology is similar to that of AciCC, differentiation between SC and AciCC is difficult using HE staining alone. Bishop et al reported that 19% of parotid gland AciCC cases were SC.^[7]^ Similarly, nine of the 23 cases with oral minor salivary gland-originated SC were initially diagnosed as AciCC, suggesting that the differentiation between SC and AciCC is difficult. In our cases, immunostaining revealed that the tumor was positive for CK19, S-100, vimentin, mammaglobin, GCDFP15, GATA3, and MUC4. These markers have been reported to be useful for differentiating SC from other salivary gland tumors.^[4,13–17]^ The results were consistent with the findings frequently observed in the previously reported cases of SC, thereby facilitating differentiation (Tables 1 and 3^[4]^). However, these immunohistochemical findings are not uniform in all SC and AciCC cases; therefore, it is essential to confirm the presence of the ETV6-NTRK3 fusion gene by genetic analysis to establish a definite diagnosis.^[4,19,38]^ Thus, an accurate diagnosis of SC can be established by the sequential use of hematoxylin and eosin histological screening followed by immunohistological investigation and genetic analysis. Surgical resection was performed as the initial treatment in all 23 patients. Local recurrence was noted in three cases, which may have been due to surgical margin positivity in two cases and a close margin in one case. As the histopathological findings of excisional biopsy revealed a close margin in case 1, additional resection was performed with a 10-mm safety margin to prevent local recurrence. Although most SC is considered a low-grade malignancy, additional resection should be performed to secure a safety surgical margin in positive cases and cases with a margin close to the tumor to prevent local recurrence. Seventeen of the 23 cases with oral minor salivary gland-originated SC were treated at cT1N0, that is, in the early stage. Late cervical lymph node metastasis to cervical lymph nodes developed in three cases (14.3%) and seven years after surgery in one case. The frequency of cervical lymph node metastasis is higher in SC than in AciCC: 8–11% in AciCC^[44]^ and approximately 25% in SC.^[6,22,23,40,45,46]^ Sethi et al^[6]^ reported that many cases of intercalated duct-type cell-predominant AciCC are metastatic, and these are highly likely to be SC, thereby confirming that the frequency of cervical metastasis may be higher in SC than in AciCC. Therefore, long-term post-operative observation may be necessary for SC cases considering the possibility of late cervical lymph nodes metastasis. In general, most SCs are considered low-grade malignancies, and the treatment outcome is favorable.^[11]^ This was supported by the fact that all 23 patients with oral minor salivary gland-originated SC survived for four months to nine years. However, a few patients with parotid gland-originated SC developed distant metastasis and died, suggesting a slightly poor outcome, and cases of high-grade transformation containing a highly malignant tumor component with poor outcomes have similarly been reported.^[23]^ Furthermore, the possibility of differences in the disease-free survival time among AciCC cases has been previously suggested.^[11]^ Therefore, differentiation between the two carcinoma types is important. To evaluate true malignancy and treatment outcomes of oral minor salivary gland-originated SC and AciCC, re-investigation of the previous cases diagnosed as AciCC may be necessary. SC is considered an NTRK fusion-positive cancer, together with SC of the breast and infantile fibrosarcoma.^[47]^ When the normal NTRK gene is fused with another gene to form an NTRK fusion gene, the tropomyosin receptor kinase (TRK) fusion protein is produced, which continuously activates the phosphoinositide phospholipase Cɣ, Mitogen-activated protein kinase, and Pl3K signal transmission pathways and promotes cancer cell proliferation.^[47,48]^ More recently, Skalova et al reported VIM-RET gene fusion in SC,^[49]^ and this finding may further expand the molecular definition of SC.

**Table 2:** Reported cases of secretory carcinoma of oral region.

Patient no.	Author (year)	Age	Sex	Location	Size (mm)	Stage at time of Diagnosis	Surgical Margins	Metastasis (yr, mo)	Local Recurrence (y,mo)	Treatment	Follow-up (yr, mo)	Outcome
1	Skalova et al. 2010^[4]^	51	F	Buccal mucosa	10	T1N0M0	NA	No	No	Excision	4 yr	NED
2		32	M	Upper lip	10	T1N0M0	NA	LN 7 yr 2 mo	No	Excision, re-excision ND+RT for LN metastatis	9 yr, 5 mo	NED
3		48	M	Soft palate	15	T1N0M0	NA	No	No	Excision	6 yr	NED
4	Kratochvil et al. 2012^[17]^	48	F	Upper lip	10	T1N0M0	NA	No	No	Excision	8 mo	NED
5		52	M	Lower lip	7	T1N0M0	NA	No	No	Excision	4 mo	NED
6	Griffith et al. 2013^[18]^	51	M	Buccal mucosa	21	T2N0M0	NA	No	No	Excision, ND	4 mo	NED
7	Laco et al. 2013^[19]^	34	F	Upper lip	15	T1N0M0	Negative	No	No	Excision	1 yr, 3 mo	NED
8	Luo et al. 2014^[9]^	41	F	Hard palate	4	T1N2bM0	NA	No	No	Excison, ND+RT	10 mo	NED
9	Helkamaa T et al. 2015^[20]^	35	M	Hard palate	20	T2N0M0	Negative	No	No	Excision	1 yr, 6 mo	NED
10	Aizawa et al. 2015^[10]^	41	M	Lower lip	15	T1N0M0	NA	LN 2yr	No	Excision, ND (for LN metastatis)	6 yr	NED
11	Majewska et al. 2015^[21]^	54	M	Hard palate	20	T1N0M0	Close	LN 4yr	Recurrence(4y)	Excision re-excision, SND,RT for local recurrence and LN metastatis	12 yr, 7 mo	NED
12	Skalova et al. 2016^[22]^	48	M	Upper lip	10	T1N0M0	Positive	No	rpT2 (2mo)	Excision, re-excision for local recurrence	9 mo	NED
13		69	F	Retromolat gingiva	6	T1N0M0	Negative	No	No	Excision	2 yr	NED
14		31	F	Buccal mucosa	10	T1N0M0	Negative	No	No	Excision	11 mo	NED
15		24	F	Buccal mucosa	10	T1N1M0	Positive	LN 2yr	Multipic 2y	Excision	2 yr, 4 mo	RD
16		62	F	Lip	10	T1N0M0	Negative	No	No	Excision	3 yr	NED
17	Hindocha et al. 2017^[23]^	27	F	Upper lip	24	T1N0M0	Positive	No	No	Excision re-excision for positive margin	9 mo	NED
18	Bissinger et al. 2017^[24]^	34	M	Oral floor	8	T1N0M0	NA	No	No	Escision, ND	2 yr, 4 mo	NED
19	Kai et al. 2017^[25]^	58	M	Buccal mucosa	30	T2N0M0	NA	No	No	Excision	1yr	NED
20	Boliere et al. 2019^[26]^	57	M	Hard palate	20	T2N0M0	Negative	No	No	Excision, ND	3 yr	NED
21	Paudel et al. 2019^[8]^	54	F	Buccal mucosa	10	T1N0M0	NA	No	No	Excision	2 mo	NED
22	Present case.1	65	F	Upper lip	15	T1N0M0	Close	No	No	Excision, re-excision for close margin	5 yr	NED
23	Present case.2	84	M	Buccal mucosa	17	T1N0M0	Negative	No	No	Excision	2 yr	NED

LN = lymph node, NA = not available, ND = neck dissection, NED = no evidence of disease, RD = residual disease, RT = radiation therapy, SND = selective neck dissection.

**Table 3:** Summary of immunohistochemical studies [based on Skálová et al. 2010].

IHC Marker	SC, (%)	AciCC, (%)
CK19 (+)	15/15 (100)	2/10 (20)
S-100 protein (+)	15/15 (100)	4/12 (33)
Vimentin (+)	15/15 (100)	3/12 (25)
Mammaglobin (+)	22/25 (88)	1/19 (5)
GCDFP-15 (+)	8/11 (73)	4/10 (40)
MUC4 (+)	9/11 (82)	0/8 (0)

CK19 = cytokeratin 19.

Entrectinib is a potent inhibitor of TRK A, B, and C, which has been shown to elicit anti-tumor activity against NTRK gene fusion-positive solid tumors, including SC. The effectiveness of entrectinib was recently demonstrated in the studies of tumor alterations responsive to targeting receptor kinases-2 involving patients with NTRK fusion-positive cancer; five of the six patients with SC equally responded to the treatment. Entrectinib inhibits the phosphorylation of the TRK fusion protein, which in turn inhibits its downstream signal transmission and consequently results in the inhibition of cancer cell proliferation.^[47,48,50,51]^ Thus, it may be a useful treatment option for patients in whom surgery is not indicated and those with distant metastases.

## 4 Conclusion

We reported two patients with oral cavity-originated SC and performed a pooled analysis of previously reported SC cases. It is highly likely that many cases of SC were previously diagnosed as AciCC owing to their similar histological findings. The treatment strategy for minor salivary gland-originated SC is similar to that for AciCC; however, SC is often highly malignant, resulting in a high risk of cervical lymph node metastasis. According to these results, establishing an accurate diagnosis together with pathologists and confirming the ETV6-NTRK3 fusion gene by genetic analysis is important.

## Acknowledgments

The author would like to thank Editage (www.editage.com) for English language editing.

## Author contributions

MO contributed to the conception and design, acquisition of data, analysis, and interpretation of data. TY, KS, TS, JK and TM contributed to analysis of the patient's data/findings. MS carried out the immunoassays and immunohistochemical staining. SY conceived the study, participated in its design and coordination, and helped to draft the manuscript. All authors read and approved the final manuscript.

**Data curation:** Takahiro Yamaguchi, Keisuke Suzuki, Mai Seki-Soda, Takahiro Shimizu, Jun Kurihara, Takaya Makiguchi.

**Project administration:** Satoshi Yokoo.

**Writing – original draft:** Masaru Ogawa.

**Writing – review & editing:** Masaru Ogawa.
